# Joint Reconstituted Signaling of the IL-6 Receptor via Extracellular Vesicles

**DOI:** 10.3390/cells9051307

**Published:** 2020-05-24

**Authors:** Philipp Arnold, Wiebke Lückstädt, Wenjia Li, Inga Boll, Juliane Lokau, Christoph Garbers, Ralph Lucius, Stefan Rose-John, Christoph Becker-Pauly

**Affiliations:** 1 Anatomical Institute, Christian-Albrechts-University Kiel, Otto-Hahn Platz 8, 24118 Kiel, Germany; w.lueckstaedt@anat.uni-kiel.de (W.L.); w.li@anat.uni-kiel.de (W.L.); rlucius@anat.uni-kiel.de (R.L.); 2 Biochemical Institute, Christian-Albrechts-University Kiel, Otto-Hahn Platz 9, 24118 Kiel, Germany; ingaboll@bmb.sdu.dk (I.B.); rosejohn@biochem.uni-kiel.de (S.R.-J.); 3 Institute of Pathology, Otto-von-Guericke University Magdeburg, Leipziger Str. 44, 39120 Magdeburg, Germany; juliane.lokau@med.ovgu.de (J.L.); christoph.garbers@med.ovgu.de (C.G.); 4 Department of Biochemistry and Molecular Biology, University of Southern Denmark, Campusvej 55, 5230 Odense M, Denmark; 5 MSH Medical School Hamburg, Am Kaiserkai 1, 20457 Hamburg, Germany

**Keywords:** extracellular vesicles, IL-6 receptor signaling, chemokine

## Abstract

Interleukin-6 (IL-6) signaling is a crucial regulatory event important for many biological functions, such as inflammation and tissue regeneration. Accordingly, several pathological conditions are associated with dysregulated IL-6 activity, making it an attractive therapeutic target. For instance, blockade of IL-6 or its α-receptor (IL-6R) by monoclonal antibodies has been successfully used to treat rheumatoid arthritis. However, based on different signaling modes, IL-6 function varies between pro- and anti-inflammatory activity, which is critical for therapeutic intervention. So far, three modes of IL-6 signaling have been described, the classic anti-inflammatory signaling, as well as pro-inflammatory trans-signaling, and trans-presentation. The IL-6/IL-6R complex requires an additional β-receptor (gp130), which is expressed on almost all cells of the human body, to induce STAT3 (signal transducer and activator of signal transcription 3) phosphorylation and subsequent transcriptional regulation. In contrast, the IL-6R is expressed on a limited number of cells, including hepatocytes and immune cells. However, the proteolytic release of the IL-6R enables trans-signaling on cells expressing gp130 only. Here, we demonstrate a fourth possibility of IL-6 signaling that we termed *joint reconstituted signaling* (JRS). We show that IL-6R on extracellular vesicles (EVs) can also be transported to and fused with other cells that lack the IL-6R on their surface. Importantly, JRS via EVs induces delayed STAT3 phosphorylation compared to the well-established trans-signaling mode. EVs isolated from human serum were already shown to carry the IL-6R, and thus this new signaling mode should be considered with regard to signal intervention.

## 1. Introduction

**Figure 1:**
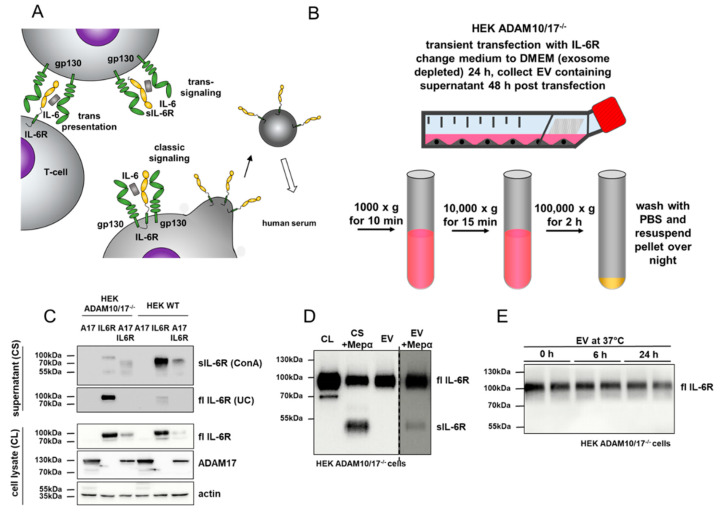
Preparation of extracellular vesicles (EVs) from cell supernatants. (**A**) Schematic overview of the known cytokine signaling modes of the IL-6/IL-6R/gp130 complex. Classic signaling occurs on cells expressing the IL-6R, whereas a soluble shed IL-6R is required for trans-signaling. Full-length IL-6R (flIL-6R) can be found in human and murine serum present on EVs. (**B**) Preparation of EVs from cell culture supernatants includes multiple centrifugation steps and the final EV pellet was dissolved in the buffer overnight to minimize vesicle destruction. (**C**) Western blot analysis of full-length IL-6R (fl IL-6R) on EVs in the presence or absence of a shedding protease (here ADAM17; A17). Note the strong reduction of flIL-6R on EVs when ADAM17 is present (HEK293 WT). ConA, Concanavalin A precipitation; UC, ultracentrifugation. (**D**) Western blot analysis of EVs isolated from cell supernatants (CS) in the presence or absence of the soluble sheddase meprin α (Mepα) for determination of the receptor orientation. Notice that flIL-6R can be shed from isolated EVs by meprin α (CL = cell lysate; CS +Mepα = ConA precipitation of cell supernatants of cells incubated with meprin α). (**E**) Incubation of isolated EVs at 37 °C for 24 h demonstrates the stability of the full-length IL-6R on EVs. No shedding product was observed.

Blockade of IL-6 signaling is a promising strategy for the treatment of chronic inflammatory conditions such as rheumatoid arthritis [1]. Binding of IL-6 to its α-receptor (IL-6R) leads to the recruitment of glycoprotein 130 (gp130), which is the signal-transducing β-receptor [2]. Crucial for the understanding of IL-6 biology is the discovery of three different signaling modes that can be induced by IL-6 and subsequent STAT3 phosphorylation (summarized in [3]): (i) Classic signaling refers to cells expressing the IL-6R together with gp130; (ii) Trans-signaling depends on the proteolytic release of the IL-6R (a process called ectodomain shedding), which in complex with IL-6 can bind and stimulate gp130 on cells lacking the IL-6R; and (iii) Trans-presentation, where, in the presence of IL-6, the membrane-bound IL-6R on one cell can bind gp130 on another cell in close proximity (Figure 1A). These different ways of IL-6 signaling enable associated cellular differentiation and proliferation in almost all cells of the body, due to the nearly ubiquitous expression of gp130.

Importantly, the three modes of IL-6 signaling are associated with different physiological and pathological conditions. Classic signaling was demonstrated to be important for macrophage polarization/differentiation from M1 to M2 state and acute phase response [4,5,6]. This is a rather anti-inflammatory function of IL-6 since only M1 macrophages were shown to exhibit pro-inflammatory activity. In bone homeostasis, IL-6 classic signaling is capable of activating osteoblasts to induce expression of the receptor activator of nuclear factor-κB ligand (RANKL) [7]. Bone resorption, on the other hand, is mediated by IL-6 trans-signaling acting on osteoclasts [8]. Several chronic inflammatory conditions are driven by IL-6 signaling [9,10,11,12]. However, due to the diversity of such diseases, it is still under investigation which IL-6 signaling mode is most relevant under which condition. Recently, it was demonstrated that activated T_H_17 cells, which lack the IL-6R, can respond to IL-6 through trans-presentation of the IL-6R by dendritic cells that are in close proximity and additionally interact via the T cell receptor [13].

The different modes of IL-6 signaling relevant for different pathological conditions have remarkable consequences for suitable therapeutic strategies targeting IL-6 signaling. To date, two monoclonal antibodies targeting either IL-6 (Siltuximab) or the IL-6R are approved for the treatment of the autoimmune Castleman disease [14] and different forms of arthritis (Tocilizumab) [15] or rheumatoid arthritis (Sarilumab) [16,17,18]. Of note, these antibodies do not differentiate between the three modes (classic-, trans-, and trans-presentation) of IL-6 signaling. To circumvent this and to address trans-signaling exclusively, a chimeric soluble gp130Fc protein (Olamkicept) was designed that can bind the soluble IL-6R to inhibit its activity on other cells but does not block the classic signaling of IL-6 [19]. Olamkicept is currently in phase IIa clinical trials for the treatment of inflammatory bowel disease. The future will show which pathological condition is most druggable by these different biopharmaceuticals. However, a profound understanding of the different modes of IL-6 signaling for certain diseases is required.

It has been known for some time that a full-length version of the IL-6R is present on extracellular vesicles (EVs), which may represent a mixture of exo- and ectosomes in human and murine serum [20,21]. However, the biological relevance of this receptor population remained unclear. We now show that this full-length membrane-bound IL-6R on EVs can be transported to and fused with cells that lack the IL-6R. This mode of signaling, termed joint reconstituted signaling (JRS), consequently enables delayed classic-signaling on those cells that otherwise would only respond to trans-signaling or trans-presentation of the IL-6R. We postulate the herein identified JRS pathways as an additional mode of IL-6 signaling that should be considered for certain (patho)physiological conditions, which could not be clearly associated with one of the other three reported signaling modes.

## 2. Materials and Methods

### 2.1. Transient Cell Transfection, Cell Lysis, and Western Blot

Transient transfection, cell lysis, and western blotting were performed as described before [22,23]. In short, cells were grown to 60–70% confluence and DMEM (Dulbecco’s Modified Eagle’s Medium) high-glucose medium (Gibco^TM^, Carlsbad, CA, USA) was changed before the addition of the transient transfection mixture. This contained (for a 10 cm dish) 5 µg DNA plasmid and 15 µL Polyethylenimin (PEI, Sigma-Aldrich, Darmstadt, Germany) in 300 µL medium without FBS (fetal bovine serum; PANBiotech, Aidenbach, Germany) incubated at 37 °C for 30 min. After 5 h, the medium was changed again to remove PEI from the cells. To prepare cell lysates, cells were harvested using a cell scraper (Sarstedt, Newton, NC, USA) and transferred onto ice. After washing the cells with ice-cold PBS (phosphate-buffered saline) three times, lysis-buffer (1% Triton X-100, Complete protease inhibitor cocktail (Roche, Penzberg, Germany) in PBS) was added for 30 min. Cell lysates were centrifuged at 13,000× *g* for 15 min at 4 °C to remove cell debris. The supernatant was then mixed with SDS (sodium dodecyl sulfate)-containing loading buffer, and proteins were denatured for 10 min at 98 °C. After separation by SDS-PAGE proteins were transferred to an Immobilon^®^-FL PVDF (Merck Millipore, Burlington, MA, USA) or nitrocellulose 0.2 µm (GE Healthcare Amersham™ Protran™ NC, GE Healthcare, Boston, MA, USA) membrane, blocked with 5% milk or BSA (bovine serum albumin) (pSTAT3 experiments) in TBS (Tris-buffered saline) (100 mM Tris-HCL, 685 mM NaCl, pH 7.5) for 2 h and then incubated with the primary antibody overnight at 4 °C (Table 1). Afterward, the membranes were washed with TBS-T (TBS, 0.25% Tween 20, 1% Triton X-100, pH 7.5), and the appropriate secondary antibody was added for 1 h. The membranes were washed again with TBS-T and were then developed using a LAS 3000 mini (FujiFilm, Germany) or Amersham^TM^ Typhoon^TM^ Biomolecular Imager infrared imaging system (pSTAT3 experiments). Concanavalin A (Sigma Aldrich, Darmstadt, Germany) (ConA) precipitation from cell culture supernatants was performed as described [22].

### 2.2. Purification of IL-6 Receptor Containing EVs From Cell Culture Supernatants

To reduce shedding of the IL-6 receptor (IL-6R) from the cell surface HEK293 cells deficient for ADAM10/17 [24] were used to express different IL-6R constructs. Twenty four h post-transfection medium was changed to exosome depleted (Thermo Scientific), and an additional 24 h later, cell culture supernatants were collected. As a control for successful transfection, cells were harvested, lysed, and Western blots for the identification of relevant proteins were performed. To purify the EVs, supernatants were centrifuged at 1000× *g* for 10 min to remove cells. Subsequently, the pellets were discarded and the supernatants were centrifuged at 10,000× *g* for 15 min to remove cell debris. The pellets again were discarded and the supernatants centrifuged at 100,000× *g* for 2 h in the ultracentrifuge to spin down EVs (pellet). Afterward, the EVs were washed with PBS and the centrifugation was repeated; the supernatants were discarded carefully, and PBS was added to the pellet for resuspension at 4 °C overnight on a shaking platform. This was, in our hands, the best protocol to gain intact EVs as examined by transmission electron microscopy.

### 2.3. Transmission Electron Microscopy of EV and Cells

Negative staining transmission electron microscopy (TEM) was performed as described before [25]. The EV-containing solution was applied to negative-glow discharged continuous carbon grids (Science Service, Munich, Germany). After 10 s, the access volume was removed using filter paper. Grids were then incubated with a half-saturated solution of uranyl acetate and removed immediately. Grids were air-dried and subsequently transferred into an JEOL 1400 Plus TEM (JEOL Germany, Munich, Germany) operating at 100 kV. The images were taken on a F416 4kx4k digital camera (TVIPS, Munich, Germany).

To image cells incubated with isolated EVs, TEM cells were grown on glass cover slips, and after the addition of EV-containing solution for 30 min, they were fixed using 3% glutaraldehyde in phosphate buffer (0.1 M phosphate in H_2_O_dest_, pH 7.4). Cells were dehydrated on cover slips, post-fixed with 2% OsO_4_, and embedded on glass slips in araldite resin using standard procedures. Using liquid nitrogen, glass slips were blasted off, and cells remained in the raisin. After ultra-thin sectioning of samples, they were contrasted using uranyl acetate and lead citrate. TEM imaging was performed as described above.

### 2.4. Confocal Laser Scanning Microscopy, z-Stacking, and Imaging

Confocal laser scanning microscopy (CLSM) was performed on live cells to enable interaction with IL-6R containing EVs. Therefore, cells were seeded into a life cell imaging chamber (Ibidi, Gräfeling, Germany), and isolated EVs containing IL-6R-GFP (fusion construct of full-length IL-6R with C-terminal GFP (green-fluorescence protein)) were added. The GFP signal could be followed in the green channel of the CLSM (FV1000, Olympus, Hamburg, Germany). After EVs attached to cells, z-stacking images were taken under green and white light channels. The obtained tif image stacks were converted into 3D mrc format (tif2mrc, embedded in IMOD) and imaged using the UCSF Chimera [26]. For internalization studies of GFP from vesicles, GFP and IL-6R-expressing vesicles were purified (as described above) and applied on HeLa cells previously seeded on glass coverslips in a six-well plate. After two hours of incubation, cell membranes were stained with CellBrite^TM^ Fix640 (Biozol GmbH, Germany), and 15 min later, cells were fixed using 4% Paraformaldehyde (PFA). Nuclear staining was performed using Bisbenzimide prior to visualization using CLSM.

### 2.5. Ba/F3 gp130 Cell pSTAT3 Experiments

The Ba/F3 gp130 cell line was cultivated as described before [27]. One million Ba/F3 gp130 cells were used in every experiment. Prior to stimulation, the cells were washed with PBS, starved for 2 h in a medium without FBS and Hyper-IL-6 to ensure de-phosphorylation of STAT3. After the addition of stimuli (EVs (100 µL) and IL-6 (100 ng/mL) or Hyper-IL-6 (100 pg/mL for control), incubated for the given time at 37 °C and 400 rpm, immediately pelleted (500× *g* for 3 min, 4 °C) and lysis buffer was added, as described above, including PhosSTOP™ (Sigma-Aldrich, St. Louis, MO, USA). After lysis for 30 min on ice, samples again were centrifuged at 15,000× *g* for 15 min, 4 °C and supernatant was used for further protein determination and western blot analysis.

### 2.6. Statistical Analysis

Statistical analysis was performed using GraphPad 8.0., and statistical significance was tested using two-way analysis of variance (ANOVA), and significance was assumed for ** = *P* < 0.01).

## 3. Results

### 3.1. Full-Length IL-6 Receptor is Present on Extracellular Vesicles and Prone to Ectodomain Shedding

It has previously been shown that full-length IL-6R is circulating in human and murine serum on EVs [20,21] (Figure 1A). The exact entity of these vesicles is unknow, and it is unclear whether exosomes or ectosomes or both are present [28]. We could previously show that IL-6R containing EVs are released by cells deficient for the two main sheddases ADAM10 and ADAM17 [22]. Although present in human and mouse, the function of this full-length receptor and its biological activity has not been determined. To address this issue, we established a protocol based on sequential centrifugation to purify EVs from supernatants of HEK293 cells transiently transfected with the human IL-6R (Figure 1B). To prevent shedding of the receptor by endogenous proteases, we used ADAM10/17 deficient HEK293 cells and compared the amount of EVs with wild type (WT) HEK293 cells. Therefore, supernatants were ultra-centrifuged (UC) to extract the EV fraction and then ConA precipitated to extract all glycosylated proteins from the supernatants, which include the remaining soluble IL-6R. In ADAM10/17 deficient cells, a prominent signal of full-length IL-6R was detected in the EV fraction that was reduced in WT cells (Figure 1C, supernatant UC). This reduction of the full-length signal of the IL-6R was also observed upon the re-transfection of ADAM17 together with the IL-6R in ADAM10/17 deficient cells (Figure 1C, supernatant UC). However, there is still a fraction of full-length IL-6R present in UC fractions from WT HEK cells, indicating a release mechanism for full-length IL-6R under steady-state conditions (Figure 1C, supernatant, UC). To successfully produce and purify large amounts of IL-6R containing EVs, ADAM10/17 deficient HEK293 cells were used, and EVs were analyzed by Western blot (Figure 1D). As the orientation of the IL-6R on EVs was unclear, we used the known sheddase meprin α and incubated it with the vesicles [22]. We found at least partial shedding of the full-length IL-6R (Figure 1D, fl IL-6R) to a soluble form of the IL-6R (Figure 1D, sIL-6R), indicating at least a partial type 1 transmembrane orientation of the receptor on vesicles. However, as the majority of the IL-6R remained at the vesicular surface after meprin α incubation in the given time, the presence of a mixture of exosomes and ectosomes in our purified EVs that carry the full-length IL-6R in different orientations is likely. Prior to functional tests, we further analyzed the stability of the full-length receptor on EVs, and therefore, incubated the purified vesicles for 24 h at 37 °C in PBS (Figure 1E). No obvious cleavage fragments appeared over 24 h neither at ~70 kDa (typical for ADAM proteases) [29,30] nor at ~55 kDa, typical for meprin proteases and cancer-associated variants of meprin β (Figure 1E) [22,31].

### 3.2. Isolated IL-6R Carrying Extracellular Vesicles Interact With the Plasma Membrane of Living Cells

**Figure 2:**
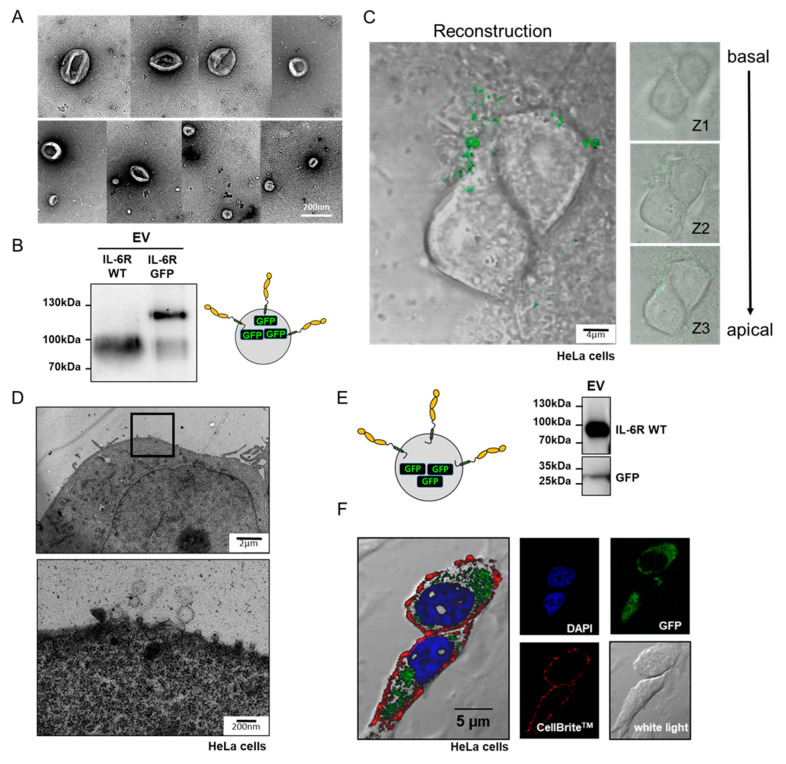
Isolated EVs interact with cells. (**A**) Transmission electron microscopic (TEM) image of EVs prepared from supernatants of transfected HEK293 ADAM10/17^−/−^ cells. (**B**) Western blot analysis of EVs containing either untagged IL-6R (IL-6R WT) or IL-6R C-terminally tagged with green-fluorescence protein (IL-6R GFP). (**C**) Z-stacking reconstruction using confocal microscopy images of EVs containing IL-6R-GFP that were added to HeLa cells. (**D**) TEM image of an ultrathin cut HeLa cell incubated with EVs containing the IL-6R. EVs interacting with the cell surface could be observed at higher magnification. (**E**) Control Western blot of EVs carrying the IL-6R and a soluble version of GFP. (**F**) Confocal microscopy image of HeLa cells incubated with vesicles depicted in E. The cell nucleus is stained in blue (Bisbenzimide), the cell membrane is stained using CellBrite^TM^ (red), GFP is in green, and a white light image was taken to display cell contours.

To show the integrity of purified EVs from supernatants of IL-6R expressing cells, we used negative staining transmission electron microscopy and observed spherical vesicles from approximately 50 nm to 200 nm in diameter (Figure 2A). This is in line with previous reports where such particle sizes were described for exosomes and ectosomes [32,33]. For better visualization, we used a C-terminally GFP-tagged IL-6R construct for transfection of ADAM10/17 deficient HEK cells and purified EVs (Figure 2B). These GFP-positive vesicles were then added to HeLa cells and after 30 min incubation, z-stacking analysis was performed using confocal microscopy (Figure 2C). We found that green fluorescent IL-6R-containing vesicles clustered at or fused with the cell surface of living cells. To gain a more detailed view about the surface interaction of isolated EVs and target cells, we applied IL-6R-carrying vesicles to HeLa cells and analyzed them in ultra-thin sections by transmission electron microscopy. Indeed, we could visualize EVs that directly interact with the cell surface of HeLa cells (Figure 2D). To further elucidate the interaction mechanism, we also expressed a soluble GFP together with the IL-6R as separate proteins and purified vesicles from the supernatant (Figure 2E). After incubation of the HeLa cells with these vesicles, cells were fixed and stained for the nucleus (DAPI) and the cell surface (CellBrite^TM^) and imaged in the CLSM. We found that a GFP-derived green signal was detected inside the HeLa cells (Figure 2E). This intracellular location of GFP is a strong indication that EVs fuse with the plasma membrane of the target (HeLa) cell.

### 3.3. Full-Length IL-6 Receptor on Extracellular Vesicles Induces Long-Term STAT3 Phosphorylation in Target Cells

**Figure 3:**
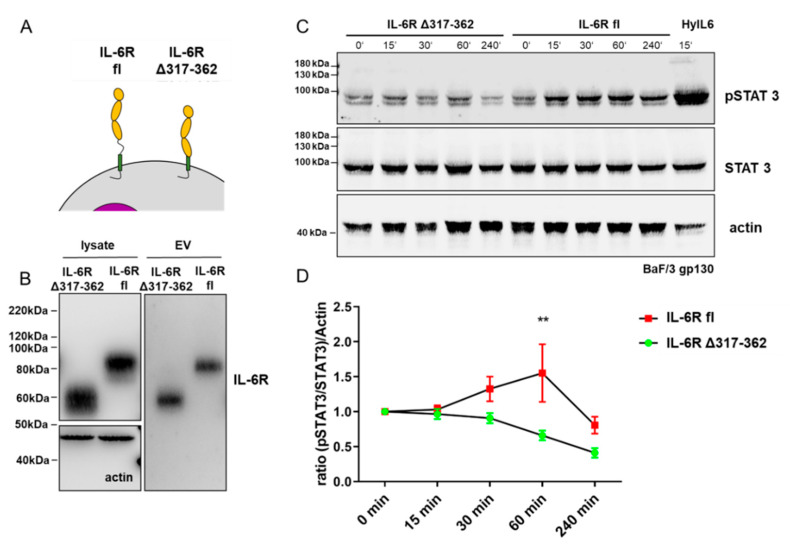
EVs containing IL-6R induces STAT3 (Signal Transducer and Activator of Transcription 3) phosphorylation (Tyr705). (**A**) Schematic presentation of full-length IL-6R (IL-6R fl) and a variant lacking the stalk region of the IL-6R (IL-6R Δ317-362). This variant was previously shown to be inactive with regard to signal transduction [38]. (**B**) Western blot analysis of cell lysate and isolated EVs carrying the full-length IL-6R (IL-6R fl) and the IL-6R missing the stalk region (IL-6R Δ317-362). The deletion variant migrates at a lower molecular mass, due to the removal of the stalk region. (**C**) Timeline experiment in BaF/3 gp130 expressing cells. These cells depend on the IL-6R and IL-6 for Stat3 phosphorylation (pSTAT3). HyperIL-6 (HyIL6), a chimeric protein of IL-6 and soluble IL-6R, was used as a positive control. Only EVs carrying the wild-type (WT) IL-6R (IL-6R fl) induces STAT3 phosphorylation. (**D**) Quantification of five independent experiments (as in C) reveals a significant increase in pSTAT3 signal in BaF/3 gp130 cells incubated with EVs carrying WT IL-6R (IL-6R fl) compared to BaF/3 gp130 cells incubated with EVs expressing the IL-6R Δ317-362 after 60 min (n = 5, statistical significance was tested using two-way analysis of variance (ANOVA), significance assumed for ** = *P* < 0.01).

The dominant signaling pathway induced by IL-6/IL-6R signaling is the JAK/STAT pathway [3], leading to the phosphorylation of STAT3 at Tyr705 and target gene expression [34,35,36,37]. To ensure that STAT3 phosphorylation is induced by vesicles carrying the IL-6R and not from some other unknown source co-purified from cell supernatants, we additionally applied EVs containing the biologically inactive truncated IL-6R Δ317-362 variant as a control (Figure 3A). Therefore, we detected a signal that migrates at a lower molecular weight in cell lysate and in the EV fraction (Figure 3B) compared to full-length IL-6R. This variant was reported previously to not form a signaling complex at the cell surface, and thus, no pSTAT3 signal was induced [38]. To measure STAT3 phosphorylation, we used the previously reported Ba/F3 cell system [27,39]. These cells express gp130 on their surface but require external IL-6R and IL-6 for signal transduction. As a positive control, a chimeric fusion protein of IL-6R and IL-6 (Hyper-IL-6) [40] was used, which is known to induce pSTAT3 in Ba/F3 cells. Upon Hyper-IL-6 stimulation (HyIL6), pSTAT3 could be detected after 15 min, which is in the expected time frame [41]. Applying EVs in a timeline experiment that carry either the IL-6R Δ317-362 or the IL-6R full-length variant revealed STAT3 phosphorylation (pSTAT3) only for IL-6R full-length EVs (Figure 3C). In repetitive experiments (n = 5), we found a significantly increased pSTAT3 signal (after 60 min) when cells were treated with EVs that had the full-length IL-6R on their surface compared to those treated with the inactive truncated version (IL-6R Δ317-362) (Figure 3D, two-way ANOVA). With these phosphorylation experiments, we can now show that the full-length version of the IL-6R has signal inducing capacity when expressed on EVs.

## 4. Discussion

IL-6 signaling via α- and β-receptors (IL-6R and gp130) is an important biological process for cell differentiation and proliferation, tissue homeostasis, and, when dysregulated, crucial for the onset and progression of certain pathological conditions, such as rheumatoid arthritis and cancer [14,15,16,17,18]. Hence, IL-6 and its receptors are therapeutic targets, and different pharmacological compounds that prevent signaling are on the market or in clinical studies. Of note, three modes of IL-6 signaling have been described, classic- and trans-signaling, and trans-presenting, which can act on the same or neighboring cells and even between distinct tissues and organs [3]. Nevertheless, not all conditions where IL-6 activity is involved could be fully addressed to one of the known signaling modes. 

**Figure 4:**
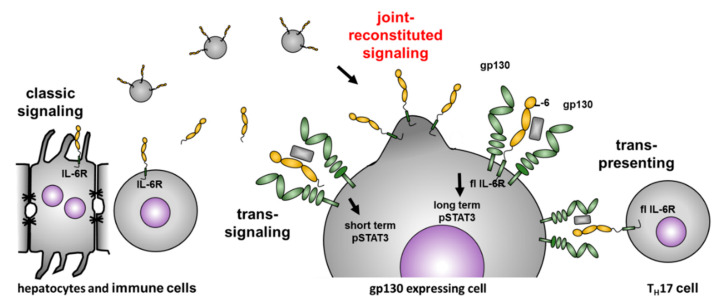
Joined reconstituted signaling of the IL-6R. The IL-6R is mainly expressed on hepatocytes and immune cells, which might be the source of EVs in serum carrying the full-length IL-6R. Our data indicate that these EVs containing the full-length IL-6R have biological activity on cells expressing gp130. The signaling properties differ from soluble IL-6R, which induces a short term (15–30 min) intracellular signaling detected via pSTAT3. The full-length receptor on EVs induces significant STAT3 phosphorylation at 60 min, and thus, generates a long-term intracellular signal.

We now introduce an additional IL-6 dependent signaling pathway, which we termed *joint reconstituted signaling* (JRS) of the IL-6 receptor (Figure 4). JRS depends on full-length IL-6R release via EVs, which can fuse with distant cells, and thus, enables long term signaling on cells that do not express the IL-6R. Similar transport/signaling modes have been described, for example, for integrins, which can be released from primary tumor cells on EVs to create a premetastatic niche in another organ [42,43,44]. Intercellular communication via EVs has been documented increasingly over the last years, not only transporting membrane-bound proteins (e.g., tetraspanins, proteoglycans, receptors, and adhesion molecules) but also cytosolic proteins, nutrients, or biologically active RNAs to regulate gene transcription in a target cell [45]. Our data demonstrate that the full-length IL-6R on isolated EVs can induce STAT3 phosphorylation via gp130 on distant cells. Most likely, EV fusion with the membrane leads to the presentation of the IL-6R on the cell surface. As considerable amounts of IL-6R were described on EVs isolated from human plasma [20], one can assume that the herein described mode of IL-6 signaling (JRS) could have physiological relevance. It would also contribute to the buffer system that regulates the level of free IL-6 in blood, which is predominantly built by proteolytically generated soluble IL-6R [46]. JRS may also enable IL-6 signaling in tissues where protease-driven trans-signaling does not work, for instance, due to the lack of relevant sheddases. Interestingly, analysis of the proteome isolated from EVs revealed the presence of ADAM10/17, two potent sheddases of the IL-6R [47,48,49,50]. Therefore, it is important to further investigate, if both the IL-6R and its sheddases can be present on the very same EV, and if this would still enable shedding and release of soluble IL-6R, which could then induce trans-signaling.

## 5. Conclusions

Our findings are particularly interesting with regard to current pharmacological approaches targeting IL-6 signaling. The different modes of intervention are nicely summarized by Garbers and colleagues [3], which emphasizes that one should carefully consider whether inhibition of classic- or trans-signaling is the best therapeutic option for the individual patient. Our findings demonstrate that under certain conditions, it might be beneficial to apply combinatorial treatment to target JRS as well. However, the importance of JRS for different pathological conditions has to be investigated further, particularly employing relevant animal models.

## Figures and Tables

**Table 1:** List of primary antibodies used.

Antigen	Host Species	Dilution	Retailer
α-IL-6 receptor (4/11)	Mouse	1:1000	In house
α-STAT3 (124H6)	Mouse	1:1000	Cell Signaling
α-pSTAT3 (D3A7)	Rabbit	1:1000	Cell Signaling
α-GFP (D5.1)	Rabbit	1:1000	Cell Signaling
α-actin	Rabbit	1:5000	Sigma-Aldrich
α-myc (ADAM17-myc)	Mouse	1:1000	Cell Signaling
